# Supplementary material for: Learning curve and surgical outcome of robotic assisted colorectal surgery with ERAS program
Source: Sci Rep. 2022 Nov 29;12:20566. doi: 10.1038/s41598-022-24665-w (PMC9709162; doi:10.1038/s41598-022-24665-w)
Supplement: Supplementary file 1 — Supplementary Information 1. [file 41598_2022_24665_MOESM1_ESM.zip › LSC Raw data-Ñ[▒K/098/O_ñw╝╨░O▒Kñσ.pdf]

正式摘要(電子簽章：000000201556062)

RCC / RCC

臺中榮民總醫院

Operation Note

[redacted]

- 手術日期：2020/10/21
- 手術醫師：蔣鋒帆，CR 陳映菁，Fellow 林昌霖，R 楊捷儒，R 李研永
- 執行麻醉：VS 施涵薰
- Anesthesia：GE
- Position：Lithotomy
- Aseptic agents：Soap，Betadine Alcohol，Betadine Aqua

Diagnosis

- Pre-operative diagnosis : Malignant tumor of rectum
- Post-operative diagnosis : Ditto.

Surgical Indication

- tumor resection

Operation

- 1. Laparoscopic low anterior resection, end-to-end anastomosis, endo GIA 60-3.5mm*2 and EEA 29mm.
  Bilateral doughnut: intact, Air leak test: done, no air leak.
  2. Segmental resection of ileum(5cm)(20cm proximal to IC valve), with double barrel ileostomy at RLQ.
  3. High ligation (+) above the left colic artery.
  4. Cut margin: proximal 14cm, distal 1.5cm.
  5. Take down of splenic flexure: nil.

Finding

- 1. A 3*2cm Bromann type II tumor at low rectum, which invaded to the muscular layer, but didn't penetrate through the serosa, No enlarged lymph nodes at mesorectum,pT3N0M0.
  2. There were no synchronous polyps or tumor at resected specimen.
  3. There was no palpable liver metastasis, no visible peritoneal seeding.
  4. Severe adhesion over lower abdomen
  5. distal ileum mesentary injury due to severe adhesion

Procedure

- General endotracheal anesthesia was induced and then the patient was placed in modified Trendelenburg lithotomy position. Preoperative antibiotics were given. A Foley catheter and nasogastric tube were placed. The abdomen was prepped and draped in standard sterile fashion. A small vertical incision was made above the umbilicus and a 12mm trocar was inserted into the peritoneal cavity by open method. Peumoperitoneum was established with CO2 inflation. Laparoscopy was inserted and then another Three 5mm trocars were inserted over LLQ, LUQ and RUQ of abdomen. One 12mm trocar was inserted over RLQ. The operative findings were mentioned as above. Adhesion lysis was done. The sigmoid colon was held by grasper and the peritoneum overlying the mesentery was scored with electrocautery. The inferior mesentery artery was identified, ligated above the left colic artery with endoclips, and transected. Dissection was done above the Gerota's fascia and toward the peritoneal attachments. The colon was mobilized from retroperitoneum overlying the sacral promontory and into the pelvis. The incision was extended laterally and medially across the peritoneal reflection. The mesorectum was then mobilized. Distal descending colon and rectum were mobilized. The mesorectum was then mobilized using sharp

- dissection until the levator ani muscle was identified. It was determined a CDH 29mm could be accomondated and the proximal colon was divided. The rectum was divided with Endo GIA 60/3.5mm*2. The midline wound was enlarged and the rectum was pulled out. The distal descending colon was transected and the specimen was removed from the operative field. The anvil of EEA was applied at the distal end of the descending colon. Pneumoperitoneum was constructed again. End-to-end anastomosis was established with EEA 29mm. One extended transverse incision at 12mm trocar site of RLQ. The terminal ileum was pulled out of the abdomen. The fascia was closed with interrupted sutures of No.1 Vicryl. Segmental resection of ileum for 5cm was done due to mesentary injury, doubel barrel ileostomy was done fix with fascia by mersilk and then matured with 4-0 monocryl with nipple at proximal limb,and the rod was inserted to support the stomy. Subcutaneous tissues were closed with interrupted 4-0 eutek. and Tissue glue was used for wound. An ostomy appliance was placed. The patient tolerated the procedure well and was brought to the POR in stable condition.
- Estimated blood loss: minimum
- 器械數量: 正確
- 紗布數量: 正確

Image

- PAINT
  - colon & rectum

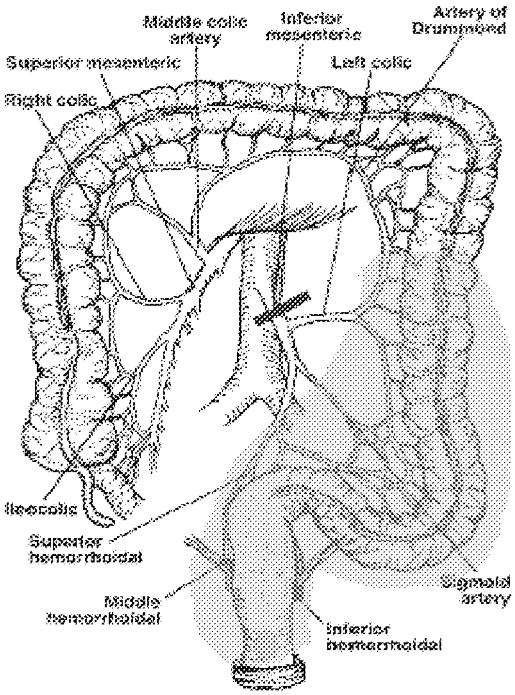

  - abdomen

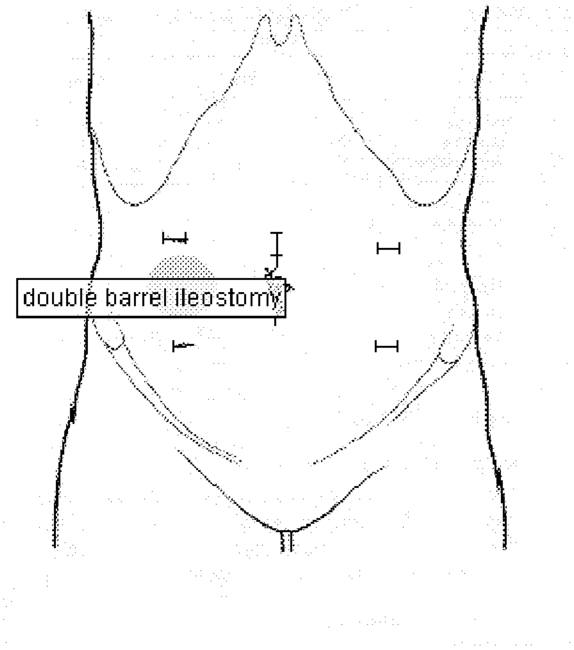

主任 2062F蔣鋒帆 / 醫師 2303K陳映菁

正式摘要(電子簽章：000000201556062)

RCC / RCC

doctorRank:B
